# Supplementary material for: Nuclear Transport of Respiratory Syncytial Virus Matrix Protein Is Regulated by Dual Phosphorylation Sites
Source: Int J Mol Sci. 2022 Jul 19;23(14):7976. doi: 10.3390/ijms23147976 (PMC9317576; doi:10.3390/ijms23147976)
Supplement: Supplementary file 1 [file ijms-23-07976-s001.zip › ijms-1770957-supplementary.pdf]

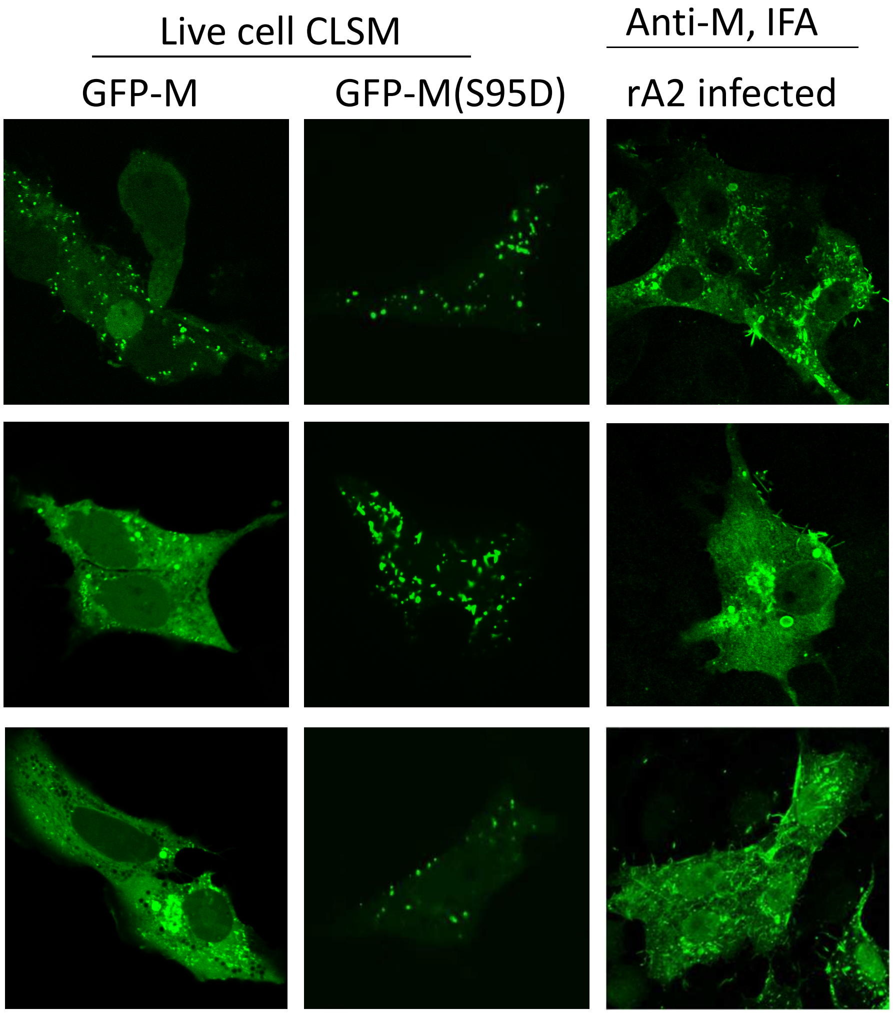

Figure S1. Aggregates of M protein in infected and transfected cells.

Images of cells transfected to express GFP-M (images on the left) or GFP-M [S95D] (images in the middle) were collected by live cell CLSM, and a selection of cells showing cytoplasmic aggregates is shown. Cells infected with rA2 were fixed at 24h p.i. and localisation of M protein analysed by immunofluorescence using a specific monoclonal antibody to M and Alexa-488 conjugated secondary antibody followed by CLSM; a selection of cells with localisation of M in virus filaments is shown (images on the right).

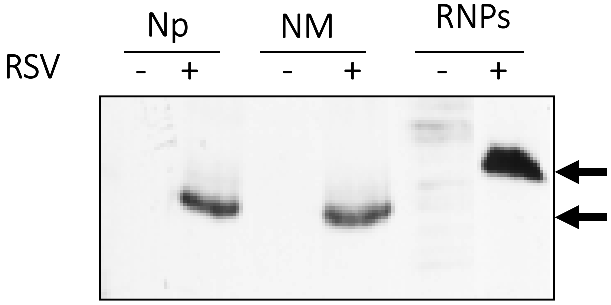

Figure S2. M protein associated with RNPs is larger than M in the nucleus.

RSV infected Vero cells were lysed at 18 h p.i. and RNPs purified from the cytoplasmic fraction as previously [7]. The nuclei were collected and fractionated into nuclear membrane and nucleoplasm as previously [10]. Samples were analysed by SDS-PAGE followed by Western blotting with specific anti-M monoclonal antibody and detection with anti-mouse IgG conjugated to HRP and enhanced chemi-luminescence. Arrows indicate M protein.
